# Global “flu-ization” of COVID-19: A perspective from Vietnam

**DOI:** 10.3389/fpubh.2022.987467

**Published:** 2022-10-03

**Authors:** Khoi Quan Nguyen, Le My Anh Nguyen, Andrew W. Taylor-Robinson

**Affiliations:** ^1^College of Health Sciences, VinUniversity, Hanoi, Vietnam; ^2^Faculty of Medicine, Imperial College London, London, United Kingdom; ^3^Center for Global Health, Perelman School of Medicine, University of Pennsylvania, Philadelphia, PA, United States

**Keywords:** COVID-19, common illness, pandemic fatigue, prevention measure, 5K policy, Vietnam

## Field statement

The widespread Omicron variant of SARS-CoV-2 causes mild to asymptomatic manifestations. Immunity is increasing among populations thanks to both vaccination and natural infection. A more relaxed attitude toward COVID-19 preventive behaviors that is becoming prevalent globally is driven by “pandemic fatigue”, a normal and expected reaction to a prolonged public health crisis. Moreover, there is a growing societal belief that as of mid-2022, SARS-CoV-2 is no more infectious than the common cold and no more virulent than influenza and should be considered like seasonal viral outbreaks. During the first 18 months of the COVID-19 pandemic, Vietnam was one of very few countries to successfully control community transmission, achieved by rigorous adherence to contact tracing, isolation, testing, and physical distancing. In this Opinion article, we contend that — at least in a Vietnamese context — it is too soon to consider COVID-19 as an endemic disease. Instead, we urge national and local measures to rekindle public support for continued, modified, implementation of recommended protective intervention as a “future-proofing” response strategy. In the short- to medium-term, this provides best practice preparedness for the possible emergence of SARS-CoV-2 variants that are more infectious and pathogenic than the currently globally predominant Omicron. This would help to control not only COVID-19 but safeguard against other infectious diseases with a similar transmission route that could pose a threat to global public health in future.

## Introduction

First authoritatively identified in Southern Africa in November 2021 (1), later substantiated by genomic surveillance data of its probable circulation as early as the previous July (2), the Omicron variant of severe acute respiratory syndrome coronavirus 2 (SARS-CoV-2) has now been established for several months as the globally predominant causative agent of coronavirus disease (2, 3). At a population level, immunity sufficient to prevent severe clinical manifestations is increasing due to the tandem effect of 70% or greater rates of second or more vaccination in most nations, excluding sub-Saharan Africa (4), and exposure to successive, distinct waves of the pandemic since the first reported outbreak in China in December 2019 (5).

In Vietnam, as with many countries that have experienced significant societally restrictive measures during the 2 previous years, the population is becoming exhausted by extended strict control policies. Mental resilience is waning rapidly, creating a profound psychological burden, particularly among individuals who were already more fragile (6). So-called “pandemic fatigue” is an expected and natural response to a prolonged public health crisis, leading to an increasingly relaxed attitude to personal preventive behaviors (7). Such demotivation in the face of currently low national COVID-19 case numbers is perhaps why public perception is growing that in the late summer of 2022 SARS-CoV-2 is no more pathogenic than the common cold and is becoming epidemically seasonal like influenza (8–11). There is a sense of diminished risk to health of the Omicron variant, especially to those who have received at least two vaccine doses. Hence, the opinion is frequently voiced internationally that by now we should adopt the same attitude to COVID-19 as we do for flu — by offering an optional annual vaccination and without adherence to self-protective practices (8, 9). This is the “flu-ization” of COVID-19.

Here, we argue that it is premature to regard COVID-19 as an endemic illness (12, 13). Rather, we advocate national and sub-national strategies to reinvigorate public support for continued, if modified, implementation of recommended protective behaviors. This would provide best practice preparedness for the possible emergence of a more infectious and pathogenic SARS-CoV-2 variant in the short- to medium-term. This “future proofing” response initiative is critical to prevent a surge in cases that would otherwise arise.

## Why COVID-19 should not yet be considered as a common illness

### Omicron is not as virulent as prior variants but should not be underestimated

SARS-CoV-2 is confirmed to have spread to all bar one of the 193 countries recognized by the World Health Organization (14). To date, the global burden of the pandemic is nearly 500 million confirmed infections and more than 6 million reported deaths (14). The Alpha variant (originally called B.1.1.7) was the first to be identified as a variant of concern, having arisen in the United Kingdom in September 2020 (2, 15). This was characterized by higher transmissibility and virulence than the wild-type single-stranded RNA virus, likely due to its numerous genetic mutations, especially in the sequence encoding the surface spike protein that binds to the host cell receptor angiotensin-converting enzyme 2, mediating viral cell entry (16, 17). For the first 1.5 years of COVID-19 — before the Delta variant was reported — the outbreak situation in Vietnam was uncomplicated and well-controlled. As of June 28, 2021, there were 15,325 confirmed cases and a week later, on 28 June, only 92 deaths were reported for the entire country (14, 18). As the virus continued to evolve, the Delta variant of concern (B.1.617.2) first circulated in India during August 2020 (2), and by July 2021 had overtaken Alpha globally in terms of transmissibility and fatalities (16, 19). Infection with those variants shared common symptoms, including headache, nausea, loss of taste and smell, fever, difficulties in breathing, and vomiting (20). In contrast, the current dominant Omicron variant (B.1.1.529) is markedly more transmissive within a community but causes milder to no symptoms in an infected healthy person. Accordingly, Omicron is associated with lower hospitalization and death rates than Delta among diverse populations (21–24). However, from available clinical and experimental data (22–24), there is no scientific rationale to make a causal link between milder manifestations and less virulent variants.

### An accumulation of mild cases could overwhelm the healthcare system

Despite causing symptoms of lesser severity, the increased transmissibility of Omicron led to a dramatic rise in case numbers in Vietnam, spiking at around 100,000 new confirmed cases per day several weeks after the lunar new year celebrations in February 2022, for which travel restrictions were substantially eased (18). Consequently, the national healthcare system has been placed under enormous strain, culminating in March-April with hospital wards filled with patients in high-risk groups, including people with underlying health conditions, the immunocompromised and the elderly (18). In this regard, Vietnam is no different to any other country impacted at various times during the pandemic by the escalating incidence, and the pressing requirement for hospitalization of severe COVID-19 cases, often at the expense of patients with other medical complications (18). In addition to the death toll directly attributable to SARS-CoV-2 infection (persons dying from or with COVID-19, typically due to such severe clinical manifestations as respiratory distress, hypoxia, and septic shock), more people than immediately prior to the pandemic are dying from non-COVID causes. Infection with SARS-CoV-2 appears to exacerbate the severity of pre-existing comorbidities, which may accelerate deterioration in health status and increase the likelihood of a fatal outcome (25, 26). Moreover, the healthcare burden from COVID-19 has prompted a redirection of resources and redistribution of staff in hospitals and community clinics. The inevitable consequence has been a shortfall in the provision of healthcare services for patients with chronic conditions that has purportedly contributed to a reported increase in non-COVID related deaths (27, 28).

### Cross-variant immunity to SARS-CoV-2 is limited but boosted by vaccination

The adaptive immune response to SARS-CoV-2 infection is not sterilizing. People can become reinfected, even after vaccination (29), usually with an isolate that is a discrete variant of the one to which they were previously exposed (30). The level and rate of attainment of herd immunity to COVID-19 achieved through natural infection are now greatly enhanced by multiple vaccination of a high proportion of a country's residents. Nevertheless, there are growing concerns regarding how best to achieve widespread attainment of acquired immune status. For instance, vaccine equity is critically important to ensure immunity across the global community. There is a huge disparity between the abundant vaccine stockpiles in high-income countries and the strictly limited supply of in-date batches of vaccines in low-income countries, notably in Africa (31). These nations have had to rely on help from non-governmental organizations and aid programs, spearheaded by the COVAX initiative, to seek access to an adequate number of doses (32). While maintaining extended lockdown as their primary preventive strategy, low-income countries must also deal with the growing issues of income inequality, poor sanitation and hygiene, mental health, and other infectious diseases such as HIV/AIDS, tuberculosis, and malaria. Each of these may have had a detrimental impact on their population prior to COVID-19 and is now complicating vaccine distribution, thereby exacerbating international disparities in the management of the pandemic (33, 34). At a sub-national level, regional differences in supply chains, public health infrastructure, and availability of skilled medical and nursing staff to roll out vaccine delivery also generate inequality between communities (35).

## Seeking publicly acceptable and easily actionable solutions

### Considering COVID-19 as a common illness — A rationale borne of influenza endemicity

Modeling the natural history of an emerging infectious disease presupposes that transmission will eventually become endemic within affected communities. When this tipping point is reached, it would be reasonable to consider COVID-19 as a common illness, much like other viral pathogens of the upper respiratory tract. In this scenario, it is anticipated that jurisdictions in all territories would consider control and prevention measures to no longer be mandatory, whereupon the vaccination rate may be predicted to drastically decline. As has been observed repeatedly concerning the seasonal influenza vaccine, past behavior is a good predictor of future attitudes toward immunization. The comparison with influenza is particularly valid for COVID-19 since vaccination compliance for these two infections belongs to the same recurring societal behavior pattern. Annual vaccination against influenza is strongly recommended for all persons 6 months and older who do not have contraindications. Nonetheless, population data have consistently shown that most people in most countries fail to attend inoculation, even if it is offered free of charge (36). A major reason for this complacency is that influenza is not perceived by healthy individuals as a serious threat. Yet, every year influenza kills millions of people worldwide, albeit typically the immunocompromised and the elderly. Without a sustained, high level of acquired immunity to SARS-CoV-2 among the community, we should anticipate that virus mutation will inevitably lead to the emergence of new variants of concern.

It is worth noting that, when compared to influenza, SARS-CoV-2 causes more respiratory complications and seems to carry a higher mortality risk, as well as affecting vulnerable populations with greater frequency (37). The hospitalization rate in France from COVID-19 during the first wave of the pandemic was nearly double that typically seen with influenza, as was the use of invasive mechanical ventilation and length of stay in the intensive care unit (34). These comparisons reinforce the inequivalence between influenza and SARS-CoV-2. Moreover, post-acute and long-COVID-19, the manifestations of which present for longer than expected even in patients with mild symptoms, is becoming a cause for concern as these persons require more medical care attention and increase the burden on the already overwhelmed healthcare system (38).

### Assessing the outbreak situation using appropriate parameters

On a positive note, two and a half years into the global battle against COVID-19 we are now equipped with accurate tools for epidemiological surveillance, improved treatments for severe disease and a deeper understanding of the pathophysiology of the virus that will enable us to combat more effectively future major outbreaks of SARS-CoV-2. In Vietnam, following the rapid surge in Omicron incidence combined with the excellent vaccination rate across all demographic groups (39), a daily case number update for each province or city is available (18). However, we contest that tracking is no longer of value as an infection control measure since it does not reflect the exact status of the pandemic in real time. The time is now ripe for the Vietnamese Ministry of Health to implement alternative ways to assess the infectious disease burden — as well as the mental health, economic and societal impact — of the next phase of the pandemic. All too frequently the statistics for hospitalized cases and deaths have been discussed as the only parameters of the enormous toll on communities that have endured long periods of isolation, limited social interaction and restricted movement. To reduce ongoing indirect harm from COVID-19, we should also identify and focus on vulnerable people, especially those who are disadvantaged by inequalities.

### Strengthening vaccination programs against SARS-CoV-2

Analysis of global data has consistently shown the effectiveness of high uptake vaccination programs in reducing the incidence of severe COVID-19. In general, the greater the vaccine coverage is, the more protection the population receives. The Vietnamese vaccination rate now exceeds the equivalent of 2.35 doses per eligible person (40). Furthermore, there is an abundance of different vaccine preparations, so demand can be met cost-effectively. We should therefore carefully consider the strategy to adopt for future implementation, including addressing the potential benefits and harm of vaccinating children in the hitherto unvaccinated 5–11 years age group, lowering vaccine booster hesitancy due to the growing perception of irrelevance, and the extent to which third or fourth doses are indeed needed among the healthy adult population.

### Long-term practice and planning for future emergencies

For long-term practice, retaining well-assimilated measures such as the Ministry of Health's established “5K policy” (including social distancing, face masks, disinfection, no gatherings, and health declarations) (19), is sustainable without exerting socioeconomic pressure or escalating mental health concerns. Of these practices, wearing a face mask and disinfecting were the easiest to accomplish but have proved efficient at blocking the transmission chain at, or close to, its source. The sustained compliance and enduring fortitude of the public has seemingly helped to control not only COVID-19 but also to reduce incidence of other infectious diseases that pose potential risks to public health. While epidemiological data are not available for Vietnam, support for this notion comes from developed countries. For instance, during the first wave of COVID-19, a marked downward trend in gastrointestinal infections was reported by the UK Health Security Agency (41). Although the drivers for this observation are multifactorial, decreased incidence of some pathogens likely resulted from the control measures and restrictions implemented in England in 2020, notably hand washing, disinfection, and physical distancing. A similar downward trend in the incidence of several major infectious illnesses, including cholera, typhoid and dysentery, was observed in China, which has implemented COVID-19 management policies similar to those in Vietnam (42). Public health approaches to outbreaks of past respiratory viral diseases such as the 2009 swine flu pandemic, where the effectiveness of identical procedures was shown in a number of countries, including China, Mexico and the USA, provide additional support for this argument (43). As Vietnam has a history and culture of public surveillance backed by military force and the population readily complies with official regulations and guidance, continuing at least some elements of the 5K non-pharmaceutical intervention policy would be tolerated in a way that is unattainable in Western societies (44). Here, in a one-party socialist state, widespread acceptance of inclusive and equitable public health policies was reinforced by initial success in controlling COVID-19 (45).

It is understandable that people the world over are becoming complacent about the COVID-19 pandemic (46). This apparent habituation of attitudes is due to a combination of factors, including: the weariness of communities to endure further restrictive measures such as extended lockdowns; the now ready access to an efficacious vaccine in most territories; and the fact that Omicron is appreciably less virulent than its predecessors as the predominant variant of SARS-CoV-2. So, it is argued, the risk to a healthy person is lowered since they will either have been vaccinated multiple times, have had a clinical episode of infection, or both. Hence, attained artificially and/or naturally, their immunity will be sufficient to combat the currently circulating pathogen in the same way that it might the common cold or influenza viruses. The perception is that COVID-19 is becoming a common illness. This is based on the frequent misperception that as Omicron is less pathogenic than Delta, if and when it is replaced by another predominant variant this will be less virulent still (47). Yet, the premise for this relaxation is not well-informed since virus mutations are random events and so are difficult to predict. If a variant of concern should arise it may be more transmissible, cause more severe disease, evade natural immunity or even be resistant to existing vaccines (48).

## Conclusion

At the end of June 2022, Vietnam detected the emergence of Omicron subvariant BA.5 (49), which is thought to be the fastest spreading subvariant recorded so far, but for which evidence of its severity is awaited. Now is not the time to declare the end of the COVID-19 pandemic, a “new normal” of predictable and mild seasonal outbreaks of SARS-CoV-2. It was only at the start of March 2022 that Vietnam was on the verge of announcing a change to endemic status (50). Within days, the country was experiencing its greatest recorded surge in COVID-19 case numbers and the enduringly resilient people were subjected to localized lockdown regulations. The pandemic remains a serious public health problem and should be considered as such—not just by epidemiologists, virologists, and clinicians—but, critically, also by those in authority.

Vietnam is opening again, tourists are returning, and the economy is growing once more. However, until such time as immunity to SARS-CoV-2 is firmly established within the community the healthcare system could yet be overwhelmed when challenged by an emerging virulent strain. In order to safeguard against such a scenario, effective epidemiological surveillance and infection control measures should be emphasized. Predictive analytics should be used to forecast virus variant spread and to estimate new case numbers, associated hospitalizations and deaths with an acceptable degree of uncertainty. Immunization with a third vaccine dose, or even a fourth for vulnerable persons, should be mandatory, while local health authorities should be watchful to reinstate 5K regulations, either in full or a modified version. As the evolution of SARS-CoV-2 is uncertain, by not easing control measures entirely Vietnam can regain its position held during the first 18 months of COVID-19 as a global leader in pandemic control (45).

## Author contributions

Article conception: NKQ and AWT-R. Literature search and data collection: NKQ and NLMA. Interpretation of information: all authors. Writing—original draft preparation: NKQ. Writing — manuscript preparation: NLMA. Writing — editing critically for important intellectual content: AWT-R. All authors read and approved the final version of the manuscript.

## Conflict of interest

The authors declare that the research was conducted in the absence of any commercial or financial relationships that could be construed as a potential conflict of interest.

## Publisher's note

All claims expressed in this article are solely those of the authors and do not necessarily represent those of their affiliated organizations, or those of the publisher, the editors and the reviewers. Any product that may be evaluated in this article, or claim that may be made by its manufacturer, is not guaranteed or endorsed by the publisher.

## References

[1] Callaway E. Heavily mutated Omicron variant puts scientists on alert. Nature. (2021) 600:21. 10.1038/d41586-021-03552-w 34824381

[2] Sharif N Alzahrani KJ Ahmed SN Khan A Banjer HJ Alzahrani FM. Genomic surveillance, evolution and global transmission of SARS-CoV-2 during 2019-2022. PLoS ONE. (2022) 17:e0271074. 10.1371/journal.pone.0271074 35913920 PMC9342790

[3] Taylor CA Whitaker M Anglin O Milucky J Patel K Pham H. COVID-19-associated hospitalizations among adults during SARS-CoV-2 Delta and Omicron variant predominance, by race/ethnicity and vaccination status – COVID-NET, 14 States, July 2021-January 2022. MMWR Morb Mortal Wkly Rep. (2022) 71:466–73. 10.15585/mmwr.mm7112e2 35324880 PMC8956338

[4] Taylor-Robinson SD Morgan MY Spearman CW Suliman AAA Corrah T Oleribe OO. Why SARS-CoV-2 vaccination still matters in Africa. QJM. (2022) 115:191–2. 10.1093/qjmed/hcac014 35080615

[5] Muralidar S Ambi SV Sekaran S Krishnan UM. The emergence of COVID-19 as a global pandemic: understanding the epidemiology, immune response and potential therapeutic targets of SARS-CoV-2. Biochimie. (2020) 179:85–100. 10.1016/j.biochi.2020.09.018 32971147 PMC7505773

[6] Xavier FP. Stress management: COVID-19 – psychological and spiritual context. J Stem Cells Regen Med. (2020) 16:92. 10.46582/jsrm.1602014 (accessed July 6, 2022). 33414586 PMC7772812

[7] World Health Organization. Regional Office for Europe. Pandemic Fatigue: Reinvigorating the Public to Prevent COVID-19: Policy Considerations for Member States in the WHO European Region. (2020). Available online at: https://apps.who.int/iris/handle/10665/335820 (accessed July 6, 2022).

[8] The New York Times. Shrugs Over Flu Signal Future Attitudes About Covid. (2022). Available online at: https://www.nytimes.com/2022/03/18/health/flu-covid.html (accessed July 6, 2022).

[9] The Times of Israel. Spain Leads Call for COVID-19 to be Treated Like Flu. (2022). Available online at: https://www.timesofisrael.com/spain-leads-call-for-covid-19-to-be-treated-like-flu/ (accessed July 6, 2022).

[10] Byun WS Heo SW Jo G Kim JW Kim S Lee S. Is coronavirus disease (COVID-19) seasonal? A critical analysis of empirical and epidemiological studies at global and local scales. Environ Res. (2021) 196:110972. 10.1016/j.envres.2021.110972 33705770 PMC7941024

[11] Li Y Wang X Nair H. Global seasonality of human seasonal coronaviruses: a clue for postpandemic circulating season of severe acute respiratory syndrome coronavirus 2? J Infect Dis. (2020) 222:1090–7. 10.1093/infdis/jiaa436 32691843 PMC7454715

[12] Chau MN; Bloomberg Asia Edition. Vietnam Mulls Shift to Covid Endemic Path as Virus Surges. (2022). Available online at: https://www.bloomberg.com/news/articles/2022-03-04/vietnam-mulls-shift-to-covid-endemic-path-even-as-virus-surges (accessed July 6, 2022).

[13] Reuters. WHO Warns Against Treating COVID-19 Like Flu. (2022). Available online at: https://www.reuters.com/business/healthcare-pharmaceuticals/who-warns-against-treating-covid-19-like-flu-2022-01-11/ (accessed July 6, 2022).

[14] World Health Organization. WHO Coronavirus Disease (COVID-19) Dashboard. Available online at: https://covid19.who.int/table (accessed July 6, 2022).

[15] Chand M Hopkins S Dabrera G Achison C Barclay W Ferguson N. Investigation of Novel SARS-CoV-2 Variant: Variant of Concern. Public Health England report, version 1 (2020). Available online at: https://assets.publishing.service.gov.uk/government/uploads/system/uploads/attachment_data/file/947048/Technical_Briefing_VOC_SH_NJL2_SH2.pdf (accessed July 6, 2022).

[16] Rajah MM Hubert M Bishop E Saunders N Robinot R Grzelak L. SARS-CoV-2 Alpha, Beta, and Delta variants display enhanced spike-mediated syncytia formation. EMBO J. (2021) 40:e108944. 10.15252/embj.2021108944 34601723 PMC8646911

[17] Zhang J Xiao T Cai Y Lavine CL Peng H Zhu H. Membrane fusion and immune evasion by the spike protein of SARS-CoV-2 Delta variant. Science. (2021) 374:1353–60. 10.1126/science.abl9463 34698504 PMC10763652

[18] Vietnam, Government. Portal of the Ministry of Health About COVID-19 Pandemic. Available online at: https://covid19.gov.vn (accessed July 6, 2022).

[19] Minh LHN Khoi Quan N Le TN Khanh PNQ Huy NT. COVID-19 timeline of Vietnam: important milestones through four waves of the pandemic and lesson learned. Front Public Health. (2021) 9:709067. 10.3389/fpubh.2021.709067 34900885 PMC8651614

[20] Centers for Diseases Control Prevention COVID-19. What You Need to Know About Variants. (2022). Available online at: https://www.cdc.gov/coronavirus/2019-ncov/variants/about-variants.html (accessed July 6, 2022).

[21] Christensen PA Olsen RJ Long SW Snehal R Davis JJ Ojeda Saavedra M. Signals of significantly increased vaccine breakthrough, decreased hospitalization rates, and less severe disease in patients with coronavirus disease 2019 caused by the Omicron variant of severe acute respiratory syndrome coronavirus 2 in Houston, Texas. Am J Pathol. (2022) 192:642–52. 10.1016/j.ajpath.2022.01.007 35123975 PMC8812084

[22] Halfmann PJ Iida S Iwatsuki-Horimoto K Maemura T Kiso M Scheaffer SM. SARS-CoV-2 Omicron virus causes attenuated disease in mice and hamsters. Nature. (2022) 603:687–92. 10.1038/s41586-022-04441-6 35062015 PMC8942849

[23] Madhi SA Kwatra G Myers JE Jassat W Dhar N Mukendi CK. Population immunity and Covid-19 severity with Omicron variant in South Africa. N Engl J Med. (2022) 386:1314–26. 10.1056/NEJMoa2119658 35196424 PMC8908853

[24] Sheikh A Kerr S Woolhouse M McMenamin J Robertson C. Severity of omicron variant of concern and effectiveness of vaccine boosters against symptomatic disease in Scotland (EAVE II): a national cohort study with nested test-negative design. Lancet Infect Dis. (2022) 22:959–66. 10.1016/S1473-3099(22)00141-4 35468332 PMC9033213

[25] Minh LHN Abozaid AA-F Ha NX Le Quang L Gad AG Tiwari R. Clinical and laboratory factors associated with coronavirus disease 2019 (Covid-19): a systematic review and meta-analysis. Rev Med Virol. (2021) 31:e2288. 10.1002/rmv.2288 34472152 PMC8646520

[26] Ng WH Tipih T Makoah NA Vermeulen JG Goedhals D Sempa JB. Comorbidities in SARS-CoV-2 patients: a systematic review and meta-analysis. MBio. (2021) 12:e03647–20. 10.1128/mBio.03647-20 33563817 PMC7885108

[27] Kelly G Petti S Noah N. Covid-19, non-Covid-19 and excess mortality rates not comparable across countries. Epidemiol Infect. (2021) 149:e176. 10.1017/S0950268821001850 34338184 PMC8365039

[28] Koh HK Geller AC VanderWeele TJ. Deaths from COVID-19. JAMA. (2021) 325:133–4. 10.1001/jama.2020.25381 33331884

[29] Reynolds CJ Pade C Gibbons JM Otter AD Lin KM Muñoz Sandoval D. Immune boosting by B.1.1.529 (Omicron) depends on previous SARS-CoV-2 exposure. Science. (2022) 377:eabq1841. 10.1126/science.abq1841 35699621 PMC9210451

[30] Primorac D Vrdoljak K Brlek P Pavelić E Molnar V Matišić V. Adaptive immune responses and immunity to SARS-CoV-2. Front Immunol. (2022) 13:848582. 10.3389/fimmu.2022.848582 35603211 PMC9114812

[31] Leach M MacGregor H Akello G Babawo L Baluku M Desclaux A. Vaccine anxieties, vaccine preparedness: perspectives from Africa in a Covid-19 era. Soc Sci Med. (2022) 298:114826. 10.1016/j.socscimed.2022.114826 35228096 PMC8848721

[32] World Health Organization. COVAX Calls for Urgent Action to Close Vaccine Equity Gap. (2022). Available online at: https://www.who.int/news/item/20-05-2022-covax-calls-for-urgent-action-to-close-vaccine-equity-gap (accessed July 6, 2022). PMC9389894 35675926

[33] Donde OO Atoni E Muia AW Yillia PT. COVID-19 pandemic: water, sanitation and hygiene (WASH) as a critical control measure remains a major challenge in low-income countries. Water Res. (2021) 191:116793. 10.1016/j.watres.2020.116793 33388470 PMC7765770

[34] Madhi SA Gray GE Ismail N Izu A Mendelson M Cassim N. COVID-19 lockdowns in low- and middle-income countries: success against COVID-19 at the price of greater costs. S Afr Med J. (2020) 110:724–6. 10.7196/SAMJ.2020.v110i8.15055 32880296

[35] Centers for Diseases Control Prevention COVID-19. COVID-19 Vaccine Equity for Racial and Ethnic Minority Groups. (2022). Available online at: https://www.cdc.gov/coronavirus/2019-ncov/community/health-equity/vaccine-equity.html (accessed July 6, 2022).

[36] Centers for Diseases Control and Prevention. Influenza (Flu). Early, Low Estimates for Flu Vaccination Coverage in Some Groups Raise Concerns at CDC. (2021). Available online at: https://www.cdc.gov/flu/spotlights/2021-2022/early-vacc-coverage.htm (accessed July 6, 2022).

[37] Piroth L Cottenet J Mariet AS Bonniaud P Blot M Tubert-Bitter P. Comparison of the characteristics, morbidity, and mortality of COVID-19 and seasonal influenza: a nationwide, population-based retrospective cohort study. Lancet Respir Med. (2021) 9:251–9. 10.1016/S2213-2600(20)30527-0 33341155 PMC7832247

[38] van Kessel SAM Olde Hartman TC Lucassen P van Jaarsveld CHM. Post-acute and long-COVID-19 symptoms in patients with mild diseases: a systematic review. Fam Pract. (2022) 39:159–67. 10.1093/fampra/cmab076 34268556 PMC8414057

[39] Our World in Data. Coronavirus (COVID-19) Vaccinations. Available online at: https://ourworldindata.org/covid-vaccinations (accessed July 6, 2022).

[40] covidvax.live: Live COVID-19 Vaccination Tracker. Available online at: https://covidvax.live/location/vnm (accessed July 6, 2022).

[41] Love NK Elliot AJ Chalmers RM Douglas A Gharbia S McCormick J. Impact of the COVID-19 pandemic on gastrointestinal infection trends in England, February-July 2020. BMJ Open. (2022) 12:e050469. 10.1136/bmjopen-2021-050469 35314468 PMC8968111

[42] Li H Ling F Zhang S Liu Y Wang C Lin H. Comparison of 19 major infectious diseases during COVID-19 epidemic and previous years in Zhejiang, implications for prevention measures. BMC Infect Dis. (2022) 22:296. 10.1186/s12879-022-07301-w 35346101 PMC8958816

[43] Cascini F Hoxhaj I Zaçe D Ferranti M Di Pietro ML Boccia S. How health systems approached respiratory viral pandemics over time: a systematic review. BMJ Glob Health. (2020) 5:e003677. 10.1136/bmjgh-2020-003677 33380411 PMC7780537

[44] Vu VT. Public trust in government and compliance with policy during COVID-19 pandemic: empirical evidence from Vietnam. Public Organiz Rev. (2021) 21:779–96. 10.1007/s11115-021-00566-w

[45] Van Tan L. COVID-19 control in Vietnam. Nat Immunol. (2021) 22:261. 10.1038/s41590-021-00882-9 33627879

[46] Petherick A Goldszmidt R Andrade EB Furst R Hale T Pott A. A worldwide assessment of changes in adherence to COVID-19 protective behaviours and hypothesized pandemic fatigue. Nat Hum Behav. (2021) 5:1145–60. 10.1038/s41562-021-01181-x 34345009

[47] Williams SN Dienes K. ‘Variant fatigue'? Public attitudes to COVID-19 18 months into the pandemic: A qualitative study. PsyArXiv [Preprint]. (2021) 1–17. 10.31234/osf.io/vam4t

[48] Callaway E. What Omicron's BA.4 and BA.5 variants mean for the pandemic. Nature. (2022) 606:848–9. 10.1038/d41586-022-01730-y 35750920

[49] Le C VN Express, International. Vietnam Detects Omicron Subvariant BA.5. (2022). Available online at: https://e.vnexpress.net/news/news/vietnam-detects-omicron-s-subvariant-ba-5-4480927.html (accessed July 6, 2022).

[50] Tuan V VN Express, International. Vietnam to Consider Covid-19 Endemic: PM. (2022). Available online at: https://e.vnexpress.net/news/news/vietnam-to-consider-covid-19-endemic-pm-4434433.html (accessed July 6, 2022).

